# Cost-benefit and extended cost-effectiveness analysis of a comprehensive adolescent pregnancy prevention program in Zambia: study protocol for a cluster randomized controlled trial

**DOI:** 10.1186/s13063-017-2350-4

**Published:** 2017-12-19

**Authors:** Amani Thomas Mori, Linda Kampata, Patrick Musonda, Kjell Arne Johansson, Bjarne Robberstad, Ingvild Sandøy

**Affiliations:** 1 Centre for Intervention Science in Maternal and Child Health, University of Bergen, P.O. Box 7804, 5020 Bergen, Norway; 2 Centre for International Health, Department of Global Public Health and Primary Care, University of Bergen, P.O. Box 7804, 5020 Bergen, Norway; 3 Muhimbili University of Health and Allied Sciences, P.O. Box 65001, 11103 Dar es Salaam, Tanzania; 4 Department of Public Health, School of Medicine, University of Zambia, Lusaka, Zambia; 5 Department of Global Public Health and Primary Care, University of Bergen, P.O. Box 7804, 5020 Bergen, Norway

**Keywords:** Adolescent pregnancy, Early marriage, School drop-out, Cost-benefit analysis, Extended cost-effectiveness analysis, Cash transfer, Catastrophic health expenditure, Cluster randomized controlled trial

## Abstract

**Background:**

Early marriages, pregnancies and births are the major cause of school drop-out among adolescent girls in sub-Saharan Africa. Birth complications are also one of the leading causes of death among adolescent girls. This paper outlines a protocol for a cost-benefit analysis (CBA) and an extended cost-effectiveness analysis (ECEA) of a comprehensive adolescent pregnancy prevention program in Zambia. It aims to estimate the expected costs, monetary and non-monetary benefits associated with health-related and non-health outcomes, as well as their distribution across populations with different standards of living.

**Methods:**

The study will be conducted alongside a cluster-randomized controlled trial, which is testing the hypothesis that economic support with or without community dialogue is an effective strategy for reducing adolescent childbearing rates. The CBA will estimate net benefits by comparing total costs with monetary benefits of health-related and non-health outcomes for each intervention package. The ECEA will estimate the costs of the intervention packages per unit health and non-health gain stratified by the standards of living. Cost data include program implementation costs, healthcare costs (i.e. costs associated with adolescent pregnancy and birth complications such as low birth weight, pre-term birth, eclampsia, medical abortion procedures and post-abortion complications) and costs of education and participation in community and youth club meetings. Monetary benefits are returns to education and averted healthcare costs. For the ECEA, health gains include reduced rate of adolescent childbirths and non-health gains include averted out-of-pocket expenditure and financial risk protection. The economic evaluations will be conducted from program and societal perspectives.

**Discussion:**

While the planned intervention is both comprehensive and expensive, it has the potential to produce substantial short-term and long-term health and non-health benefits. These benefits should be considered seriously when evaluating whether such a program can justify the required investments in a setting with scarce resources. The economic evaluations outlined in this paper will generate valuable information that can be used to guide large-scale implementation of programs to address the problem of the high prevalence of adolescent childbirth and school drop-outs in similar settings.

**Trial registration:**

ClinicalTrials.gov, NCT02709967. Registered on 2 March 2016. ISRCTN, ISRCTN12727868. Registered on 4 March 2016.

## Background

Globally, about 16 million girls aged 15 − 19 years, and 1 million aged less than 15 years, give birth every year [1]. In addition, it is estimated that around 2.0 − 4.4 million adolescent girls in developing countries undergo unsafe clandestine abortions to terminate unwanted pregnancies annually [2]. The complications arising from pregnancy and early childbirth are the second leading cause of death among adolescent girls aged 15–19 years in low-income and middle-income countries [1] and the fourth cause of death for this age group globally [3]. The risks of preterm birth and low birth-weight are higher in adolescent mothers. These also result in higher morbidity and mortality risks for the child [4, 5]. A recent study in rural Ethiopia has shown a nearly three times higher risk of death in infants born to mothers between 15 and 19 years old compared to those of mothers between 25 and 29 years [6]. The risk of infant death remains higher even when compared to infants of mothers aged between 20 and 24 years [7].

More than 30% of young girls in low-income and middle-income countries get married before reaching the age of 18 and about 14% before the age of 15 years [1]. First marriage or cohabitation is among the main causes of school drop-outs in developing countries [8]. At the same time, girls who drop out of school are more likely to engage in early sexual initiation [9] or risky sexual behaviors, or marrying and becoming pregnant, than those who stay in school and attain higher education [8, 10]. Adolescent girls in rural areas are more at risk of becoming pregnant than their urban counterparts. In Zambia, it is documented that about 36% and 20% of adolescent girls aged between 15 and 19 years in rural and urban areas have been involved in childbearing, respectively. Adolescent childbearing increases sharply from 5% among girls aged 15 years to 59% among those aged 19 years [11]. Data from the Ministry of Education, Science, Vocational Training and Early Education in Zambia shows that on average there are over 15,000 pregnancies reported each year among schoolgirls, and more than 80% of these pregnancies occur in rural areas [12].

Adolescent pregnancy is one of the greatest development challenges in low-income and middle-income countries because of its profound negative social and economic consequences. Pregnancy interrupts continuation of education, which has been shown to provide a foundation for human development [9]. Education empowers women to have greater control of their lives, participate in decision-making processes, and helps them to improve the nutritional status and health of their children and families. Pregnancy and parenthood is usually a major shock to an unprepared dependent girl who may still be in school and ignorant of basic childcare skills. As a result, many are trapped in risky sexual relationships in an attempt to cope with economic hardships [13]. Dropping out of school also reduces a girl’s future earnings, since level of education is a key determinant of income [14]. Therefore, delaying pregnancy to a more appropriate age has the potential to reduce undesirable and costly health, social and economic outcomes.

### Economic evaluations of adolescent pregnancy prevention programs

Economic evaluations inform resource allocation decisions by generating evidence about the optimal investment of scarce resources in one program or another, by comparing their costs and the potential returns for the investment. It is for this reason that economic evaluation is increasingly becoming an important criterion to inform policy and priority decisions within health systems and other sectors. A number of adolescent pregnancy prevention programs have been tested [15, 16]. However, economic evaluations of these programs are scarce [17, 18], despite the fact that they require substantial investment for scale-up. In this study, we will conduct a cost-benefit analysis (CBA) to compare costs and benefits of two adolescent pregnancy prevention package programs in Zambia. CBA, unlike other types of economic evaluation, will enable us to compare benefits across policy sectors and to capture broad program benefits from reduced adolescent birth complications and increased school completion rates.

We will also conduct an extended cost-effectiveness analysis (ECEA) that builds on the standard cost-effectiveness analysis as a quantitative method for evaluating health policies, inter-sectoral policies and policy instruments that impact population health. ECEA takes into consideration the health and financial consequences of policies including the resulting distribution of financial risk protection from catastrophic health expenditure across socio-economic groups [19]. Maternal and childcare-induced catastrophic health expenditures are common in developing countries [20, 21], despite presence of policies that advocate for free services. It is likely that a program that reduces adolescent childbearing could also prevent the catastrophic health expenditures associated with birth complications. In Zambia, surveys have shown that about 10% of households experience catastrophic health expenditures for outpatient visits alone [22]. Catastrophic health expenditure associated with adolescent childbearing are more likely to occur among the poorest households and will push them into a downward spiral of greater impoverishment thereby worsening social inequity.

## Methods

A detailed description of the trial has been published elsewhere [23]. However, we provide here a brief summary, which we think is important for understanding the planned economic evaluations to be conducted alongside the trial. The Research Initiative to Support the Empowerment of Girls (RISE) study is a three-arm cluster randomized controlled trial (cRCT), which is currently being conducted in Zambia. The trial has two intervention arms, which consist of the economic support alone and the economic support combined with community dialogue. The third arm of the trial is the control. In the RISE study, a cluster represents the catchment area for a school offering education from grade 1 to 9. In Zambia, grades 1 to 7 and grades 8 to 9 are referred to as primary school and junior secondary school, respectively.

### Study settings

The trial is being conducted in 12 districts in rural Zambia: Kalomo, Choma, Pemba, Monze, Mazabuka, Chikankata, Chisamba, Chibombo, Kabwe rural, Kapiri Mposhi, Mkushi and Luano. The districts were selected because they have medium school drop-out rates, and adolescent marriage and childbearing are common. Approximately 22% of girls in these districts have given birth by age 17 and 35% by age 18 years (Zambia Census of Population and Housing (ZCPH), 2010).

### Description of intervention packages

**Fig. 1:**
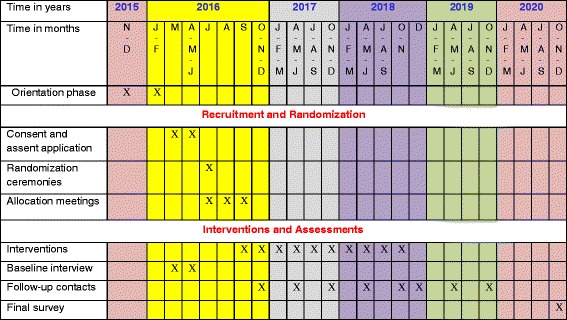
SPIRIT figure. Schedule of enrolment, interventions and assessments

The trial aims to compare the effectiveness of economic support with or without a corresponding community dialogue component, in reducing adolescent childbearing and in increasing grade 9 completion rates. The interventions were launched in September 2016 and will continue until November 2018, which is the time when the girls in the study are expected to complete grade 9 (Fig. 1). The interventions are being delivered by teachers and community health assistants (CHAs) or community health workers (CHWs). This will make the results realistic and relevant for any potential scale-up in Zambia or other similar contexts.

#### Economic support arm

In the economic support arm, the girls receive cash transfers (CT) of Zambian Kwacha (ZMW) 30 (~3 USD)/month while their parents/guardians receive ZMW 350 (~35 USD)/year. The latter amount was estimated as being sufficient to cover the costs of school uniforms and shoes, a school bag, books and writing materials. In addition, school fees are paid for the girls who enroll in grades 8 and 9 (junior secondary). The combination of the annual grant to parents and payment of school fees essentially make schooling free of cost to the families. The monthly CT are intended to give the girls the opportunity to buy things they need for themselves (e.g. lotion) instead of relying on boyfriends. A committee consisting of a teacher and two parents from the Parent-Teacher Association (PTA) distributes the payments monthly. School fees are paid directly to the school where the girls are enrolled. There is no age limit for receiving the economic support for girls are attending school; however, this support will stop after the 18^th^ birthday for girls who drop out of school.

#### Combined intervention arm

This arm combines the economic support with a community-oriented strategy. The latter involves: (1) community and parent meetings promoting supportive social norms around education for girls and postponement of early marriage and early childbearing; and (2) youth club meetings that are focused on increasing knowledge about sexual and reproductive health (SRH). It aims to change the beliefs and attitudes in the community that are related to education and marriage, and to provide information about the use of modern contraceptives among adolescent girls and boys who are either attending school or not. We expect that this strategy will delay sexual initiation and increase the use of modern contraceptives. It will reduce pregnancy among adolescent girls, and further reduce school drop-outs and delay the age of marriage as compared to providing economic support alone, and will thus also indirectly affect childbearing rates.

#### Control arm

In the control arm, the girls are offered some writing materials (exercise books, pencils and pens) as incentives to encourage them to participate in the study. This package is not likely to be sufficient to have any substantial effect on the primary or secondary trial outcomes. They are also given ZMW 20 (~2 USD) for each follow-up interview in which they participate as a token gesture to compensate them for their time. Apart from this, only standard school services and healthcare services are offered to the girls in the control clusters.

### Data collection

Data to be used in the economic evaluation will be collected alongside the main trial. Data on the status of school attendance, childbearing, birth complications and utilization of healthcare facilities are being collected by trained female research assistants through face-to-face follow-up interviews every 6 months. Cost data will be collected by the same research assistants after receiving training on the costing methodology and data collection tools. The data collection team is independent of the intervention delivery team, and the research assistants in the final interview round will be unaware of the intervention status of respondents.

### Measure of resource use and costs

#### Program costs

Program costs will be based on a detailed inventory of all resources that are used in the RISE trial. This consists of the cash support given to the girls and their parents, school fees paid by the program, costs of delivering the interventions and organizing community and youth club meetings. Other costs include salaries and benefits paid to administrative and program staff, rent and utilities, maintenance, training and other miscellaneous expenses. Research-related costs including the costs of data collection will be excluded. Data will be retrieved from expenditure invoices and receipts from the account office.

#### Educational costs

In Zambia, about 60% of girls who reach grade 7 continue to grade 8 (junior secondary school) and about 42% progress from grade 9 to 10 (senior secondary school). Drop-out rates in girls have been shown to increase from 2.6% in grade 6 to 5.3% in grade 9, which is twice the rate in boys [24]. Adolescent pregnancy is a major contributing factor for high attrition rates [12]. We expect that by reducing childbearing rates and removing the school fee barrier at junior secondary level, the program will eventually increase the number of students progressing from grade 7 to grade 8 i.e. from primary school to junior secondary school. We expect the program will also indirectly increase the transition from grade 9 to 10 i.e. junior secondary school to senior secondary school.

Shortage of classrooms is a major problem in Zambia that also hinders progress from primary to secondary school. Thus the number of places at secondary level is limited and students are often unnecessarily forced to repeat classes (and may eventually drop out of school because of frustrations), while some schools teach in shifts to accommodate all students [25]. Therefore, the RISE program will most likely create a demand for more classrooms, increased employment of teachers and additional teaching materials and furniture (such as chairs and tables etc.). We will conduct a costing study in a sample of four junior secondary schools to determine the unit cost of junior secondary education. Unit costs will help us to estimate the immediate additional educational costs of the program. Data collection tools, including a costing questionnaire, will be developed later.

#### Community and youth club meeting costs

In the combined intervention arm, we expect that there will be direct and indirect costs incurred by the participants of the community and the youth club meetings in addition to the program costs. Direct costs may include items such as travel costs to reach meeting places. Indirect costs are those associated with time lost that could have been used in economically productive activities, or in the case of the youths, it is the time that could have been used to assist at home and allow their guardians to engage in productive activities. Valuation will involve applying a minimum general wage rate for all economically productive participants. Cost data associated with participation in the meetings will be collected through a household survey that will collect consumption data for the ECEA.

### Monetary benefits for cost-benefit analysis

CBA is the only type of economic evaluation that measures expected benefits associated with a program in monetary terms. Further, CBA converts all costs and benefits into a common monetary metric that could facilitate the merging of diverse outcomes spanning different sectors. Program outcomes that will be considered for CBA include increased school completion rates and reductions in adolescent childbirth. The increase in school completion rates is expected to increase returns to education while the reduction in adolescent childbearing is expected to save healthcare costs that would otherwise have been incurred in the management of the associated birth complications.

#### Increased returns to education

By making education free and reducing adolescent childbearing rates, we expect that the RISE program will eventually increase school completion rates. According to the human capital development theory, investment in human capital i.e. education and training improves the economic productivity of an individual and hence his/her wage earning capacity. According to Mincer (1974), the logarithm of an individual’s wage earning is a linear function of schooling years combined with the quadratic function of his/her labor market experience [26]. Increased future wage earning represents the long-term benefits.

In Zambia, the rate of private returns to education was recently estimated by the Institute of Policy Analysis and Research using data from the Living Conditions Monitoring Survey (LCMS). Individuals who have completed primary school and junior secondary school were estimated to have private returns to education of 17.2% and 21.2%, respectively [27]. Thus a girl who has completed primary school will have her baseline salary increased by 17.2% and if she completes junior secondary school it will rise by 21.2%. In this study we will use average monthly incomes of 531,000 and 800,000 Kwacha, for girls who have completed primary and junior secondary education, respectively [28].

**Fig. 2:**
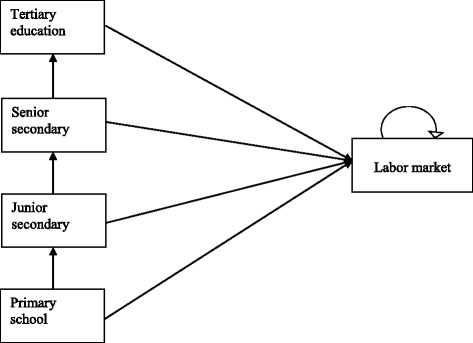
Transition states. Adolescent girls enter the model i.e. were enrolled into the RISE trial at grade 7 in primary school. At primary school and lower secondary school it is expected that a certain proportion of girls will drop out of school due to pregnancy and other reasons while the majority progress to higher levels. It is expected that the interventions provided will have direct effect of increasing the proportion of girls that progress from primary school to junior secondary and indirect effect from junior to senior secondary. It is assumed that girls dropping out of school at each level will eventually enter into the labor market where they will be paid a certain amount of wage that corresponds to their level of education

Potential lifetime earnings due to completing higher educational will be calculated using methodology similar to that of the World Bank (2009) and Chaaban (2007). This method requires construction of an age-earning profile. This is used to calculate the difference between total life earning with highest attainable level and the lowest level of education at which the girl drops out of school [29, 30]. In this study, we will use the Markov model to calculate the potential lifetime earning of the girls. We have chosen it for several reasons: first, it has the ability to model our cohorts in each arm over time to the retirement age of 60 years; second, it can calculate and discount future wage earnings efficiently, and finally, it can adequately incorporate uncertainties associated with transition probabilities between different states and earnings (Fig. 2). We will estimate the transition probabilities of moving from primary to junior secondary level and attrition rates in each arm from the trial data.

#### Averted healthcare cost

The interventions are expected to delay childbearing to a more appropriate age, hence, reducing costs associated with adolescent pregnancy and birth complications such as preterm birth, low birth-weight, eclampsia, medical abortion procedures and post abortion complications. Reduction in adolescent pregnancy and birth complications brought by the program are expected to save healthcare costs that would otherwise have been incurred by families and the healthcare system. There will also be other costs averted outside the healthcare system, which include savings from transportation costs, travelling and time loss, which may be used by the caretakers in other economically productive activities.

The data collection questionnaire that is used for face-to-face follow-up interviews at 6-month intervals includes questions about marital status, school attendance, childbearing and healthcare utilization. Girls identified to have given birth are asked extra questions to determine the incidence of preterm birth, low birth-weight, eclampsia and other complications including those related to abortion and admission of the babies to the hospital. Costing tools will be developed to estimate the provider costs of managing birth complications identified through these interviews. The costing sub-study will be conducted in four hospitals located in the study districts. We will also conduct interviews with a sample of 100 patients or caretakers at these hospitals to capture the direct costs they incur at the hospital, such as payments for medicines and consultation fees and the costs they incur outside the hospital, such as fares paid for transportation, meals and traveling time for adult caretakers. Ngalesoni et al. [31] used this approach to estimate economic costs for prevention of cardiovascular diseases in health facilities in Tanzania.

### Outcome measures for extended cost effectiveness analysis

The expected health gains and non-health gains of the program in their natural units of measurement will be evaluated in ECEA. The expected health gains are adolescent childbirths averted, and non-health gains are private/out-of-pocket (OOP) expenditures averted and financial risk protection. ECEA will also examine the disparities in health gains, OOP expenditures and financial risk protection across different socio-economic groups, and aims to assess the distributional impact of the interventions. We will use the household consumption data to rank the study populations into wealth quintiles, from the poorest to the richest.

#### Reduction in adolescent birth rates

This is the primary health outcome of the intervention, and will be expressed as the incidence of birth before a girl’s 18^th^ birthday. In comparison between the combined intervention arm and the control arm, we expect an assumed difference of -40%, and for the comparison of the economic and the control arm we expect a difference of -25%. In the comparison of the two intervention groups we expect a relative difference of 20%.

#### Private expenditures averted

As we explained earlier, the program is expected to reduce both OOP expenditures and health system costs that are associated with adolescent pregnancy and childbearing complications such as pre-term birth, low birth-weight, eclampsia, medical abortion procedures and post-abortion complications. OOP payments averted represent one of the most important non-health benefits of the program that could otherwise force households into impoverishment by reducing spending on their basic needs such as food.

#### Financial risk protection provided

The expected financial risk protection of the program is relative to income, in contrast to the absolute metric used in private expenditures averted. The underlying assumption is that $1 averted has more financial risk protection for a poor family than a rich one. We will adhere to the World Bank definition of catastrophic health expenditure, that is OOP payments for health care that exceed 10% of total household income [32] or that exceed 40% of a household’s expenditures net of food spending (household’s capacity to pay) [33]. We plan to conduct a household survey to collect household consumption expenditure data on food and non-food items including healthcare, which will help us count households with OOP payments for health that exceed the 40% threshold.

### Measuring living standards

Consumption rather than income is a preferred method for measuring standards of living in developing countries because a majority of people are employed in the informal sector, prominent production of goods at home and multiple and often constantly changing sources of income [34]. In addition, the RISE program will provide economic support to families receiving the intervention packages, and this will influence their consumption pattern. Therefore, we will conduct a household survey in order to collect consumption data that can be used to construct living standard quintiles. The data can also be used to assess levels of OOP health expenditure relative to total expenditure. Thus, the survey questionnaire will capture the consumption of food items, non-food items and consumer durables.

The category “food items” will cover both homemade food items and those purchased from the market. Non-food items will be categorized into two groups based on the frequency of purchase. Frequently purchased items will include transport, cleaning supplies, air-time vouchers, cigarettes, tobacco etc. Non-frequent non-food items will include utilities (electricity, kerosene, water, sanitation), clothes, cell-phones, health i.e. expenses on consultations and diagnostics, medicines and bed days, education (school fees, uniforms, exercise books etc.), contributions etc. Expenditures on consumer durables such as housing will be estimated using rental charges or annualized replacement costs if the dwelling is owned by the household. For frequently used items we will use a recall period of 2 weeks [34].

### Sample size estimation

Sample size calculation for the trial has been explained in detail elsewhere [23], and was based on three primary outcomes namely: incidence of births before a girl’s 18^th^ birthday, incidence of births within 8 months after the end of the intervention period, and the proportion of girls who sit for grade 9 examinations. Reduction in incidence of births and increased proportion of girls completing grade 9 are associated with healthcare cost-saving and increased returns to education, which are short and long-term benefits. The trial has a sample of 157 clusters (~4900 girls). This involves 63 in each intervention arm (1950 girls) and 31 in the control (1000 girls). It gives power of >95% to detect a difference of -40% in the “incidence of births before a girl’s 18^th^ birthday” in the combined vs control arm, power of 80% to detect a difference of -25% in the economic vs control arm and power of 70% to detect a difference of -20% in the interventions arms. For the outcome “proportion of girls who sit for grade 9 examinations”, this sample produced power of >95% to detect an assumed difference of +27% in the combined vs control arm, +15% for economic vs control and +10% between the intervention arms.

#### Sample size for economic evaluation

Several approaches to estimate sample sizes and power for trial-based cost-effectiveness analysis have been proposed in the literature [35–37]. However, they have not been applied routinely in practice, and certainly not in a cRCT. The main reason for this that is most often cited is that the sample sizes required to perform trial-based economic evaluation are usually larger than those based on clinical outcomes, something that raises both ethical and financial concerns [38]. However, the planned CBA study will facilitate the merging of diverse program benefits that are associated with different outcomes. This includes savings made in healthcare costs that are associated with reduction in incidence of births before a girl’s 18^th^ birthday and increases in returns to education associated with the increased proportion of girls who complete junior secondary school. Therefore, although the study may be underpowered, it will provide policy makers with evidence relating to the economic consequences of scaling-up each program package. Non-significant differences will be explored in the sensitivity analyses.

### Analysis

For CBA, the net social benefit (NSB) of each intervention package will be calculated by subtracting program costs from the monetary benefits. The intervention package with the best economic return will be recommended as the optimal choice for scale-up [39]. We will also calculate the benefit-cost ratio for each intervention package by dividing the incremental monetary benefits to the incremental costs relative to the control arm. All costs and benefits will be converted to present values using the commonly applied annual discount rate of 3%. Modeling of returns to investment in education will be performed in TreeAge Pro software (TreeAge Software, Inc. Williamstown, PA, USA). The net benefit will be calculated using Multilevel Models to account for clustering in the trial data [40, 41]. Net benefit will be used as an outcome variable and the intervention arm as a predictor variable. The school identification variable will be used to adjust for clustering.

For ECEA, we will use the method proposed by Xu [42], which assumes that the subsistence needs represent food spending adjusted for household size, and this is considered to be the median total household spending. Any household whose out-of-pocket health payments exceeds 40% of its capacity to pay, i.e. the total household spending less subsistence spending will be categorized as having incurred catastrophic health expenditures. ECEA calculations will be performed with the R program. We will plot concentration curves, i.e. a plot of cumulative proportions of population ranked from the poorest to the richest against cumulative proportion of adolescent births. We will also calculate concentration indices to measure levels of inequality between the trial arms.

### Sensitivity analyses

Sensitivity analyses will be performed by varying key parameters within plausible ranges to test the robustness of the results. Net benefit and cost-benefit ratio will be re-evaluated by varying program implementation costs, healthcare costs, loss in productivity, discount rates, wages and the overall change in the effectiveness of the interventions. The same will be done for the extended cost-effectiveness analysis.

### Perspective

Economic evaluations will be conducted from both the program and societal perspectives. For the program perspective we will only consider costs related to the implementation of the interventions, which could be borne by the government or any other interested organizations. For the societal perspective we will consider all costs, which include program costs, healthcare costs and household costs.

## Discussion

This paper describes a protocol for carrying out economic evaluations alongside a cRCT of a comprehensive pregnancy prevention program. The study aims to evaluate cost-benefits and extended cost-effectiveness of cash support with or without community dialogue against a null intervention. Trial-based economic evaluations are becoming increasingly popular, since decision-makers are interested to know whether the additional benefits produced by the new and more effective interventions reflect an optimal use of scarce resources, which the society could be willing to pay for.

The planned program to combat adolescent pregnancy is very comprehensive and expensive but has the potential for substantial short-term and long-term benefits in terms of improving the health, educational and economic outcomes of young girls. Another important potential benefit is the prevention of catastrophic health expenditures, which is also important to consider when determining whether such a program is worth the investment involved. Therefore, the study will generate valuable information to guide large-scale implementation of programs to tackle the problem of high prevalence of adolescent pregnancies and school drop-out in similar settings.

## Limitations

The first limitation is that the trial is investigating a very sensitive topic in a low-income setting where adolescent pregnancy and childbearing is a major problem. The sensitivity of the topic has limited the scope of questions we could ask the study participants to avoid drop-outs from the main trial. We will therefore not be able to collect cost data about pregnancy care, delivery and management of birth complications directly from the trial participants. Instead we opted to collect only the incidence data for complications that are directly associated with adolescent pregnancy. Cost data will be collected from reference cases for the identified complications, which may or may not be part of the trial.

The second limitation is that evidence has shown that morbidity and mortality due to preterm and low birth-weight is a major burden to families and to health, education and social service systems [43–45]. Preterm and low-birth-weight infants are at high risk of re-hospitalization from exposure to common childhood pathogens, anemia, relative immunodeficiency and sub-optimal nutrition following their discharge from a Neonatal Intensive Care Unit [46]. Unfortunately, the follow-up period for the trial is expected to end in 2020, at which time the majority of the babies born to participants in the RISE trial will be less than one year old. This means we will be able to collect data about incidence and reasons for re-hospitalization in pre-term and low-birth-weight babies over a short period of time only. A longer period of follow up of the infant-mother pairs would be required to capture these data and the associated costs more appropriately.

The third limitation is that the study will rely on self-reported information about the incidence of pregnancy and abortions, which are likely to be under reported in all three trial arms. This may lead to underestimation of the costs averted that would have been required to provide healthcare for complications of pregnancies that do not end in a live birth. The impact of the interventions in reducing costs associated with medical and clandestine abortions and the resulting post-abortion complications in addition depends on whether any of the trial arms are relatively more affected by the bias. The impact of differential underreporting of these outcomes on net benefits will be explored in the sensitivity analysis.

The fourth limitation is that the existing ECEA framework relies heavily on the utilization of health facilities. This means that under conditions of low utilization of healthcare services it is difficult to estimate accurately the level of catastrophic health expenditure. In Zambia, about 56% of births in rural areas take place in health facilities. This proportion decreases to about 50% among the poorest families [11]. Under this low utilization of health care, we expect low financial risk protection because the poorest families cannot afford to pay for health care.

The fifth limitation is that children born to adolescents are also more likely to have poorer life prospects than those born to adult women. Many children of adolescents perform poorly in school, and are therefore less likely to complete basic education [47], and daughters are more likely to become adolescent mothers as well [48]. Therefore, the interventions are expected to have a positive impact on the offspring’s educational attainment and future earnings, which can substantially increase the beneficial effect of the program. However, these will not be included in the analysis because of the short follow-up period.

Finally, CBA is perhaps the most challenging economic evaluation to conduct due to the fact that it requires assigning monetary values to intangible outcomes; a task that is extremely difficult. For example, adolescent pregnancy is a known risk factor for maternal morbidity and mortality, and also mortality in infants under five years of age has been estimated to be reduced by up to 10% when the mothers have received one year of education [49]. The inclusion of these impacts in CBA requires us to measure and assign monetary values to pain, suffering, emotional loss and human life in the case of premature mortality i.e. the statistical value of life, which is difficult and also debatable for ethical reasons [50]. Therefore, all intangible costs and benefits will not be included in the cost-benefit analysis [51].

## Trial status

The main protocol for the RISE trial has been published [23], and this protocol is an extension of the planned economic evaluations. At the time of submission, the trial was ongoing at the stage of delivery of interventions that will end in November 2018. The collection of cost data for economic evaluation component has not yet started.

## Abbreviations

CBA: Cost benefit analysis
CHA: Community health assistants
CHE: Catastrophic health expenditure
CHW: Community health workers
ECEA: Extended cost-effectiveness analysis
OOP: Out-of-pocket
cRCT: Cluster randomized controlled trial
RISE: Research Initiative to Support Empowerment of Girls
ZCPH: Zambia Census of Population and Housing
ZMW: Zambian Kwacha
